# Conflict, displacement and sexual and reproductive health services in Mali: analysis of 2013 health resources availability mapping system (HeRAMS) survey

**DOI:** 10.1186/s13031-015-0051-8

**Published:** 2015-09-14

**Authors:** Özge Tunçalp, Ibrahima Socé Fall, Sharon J. Phillips, Inga Williams, Massambou Sacko, Ousmane Boubacar Touré, Lisa J. Thomas, Lale Say

**Affiliations:** UNDP/UNFPA/UNICEF/WHO/The World Bank Special Programme of Research, Development and Research Training in Human Reproduction (HRP), Department of Reproductive Health and Research, World Health Organization, Geneva, Switzerland; World Health Organization, Mali Country Office, Bamako, Mali; Department of Emergency Risk Management and Humanitarian Response (ERM), World Health Organization, Geneva, Switzerland

## Abstract

**Objective:**

Little is known specifically about the effects of conflict and displacement on provision of sexual and reproductive health (SRH) services. We aimed to understand the association between levels of conflict and displacement and the availability of SRH services in post-conflict Mali.

**Methods:**

A national assessment was conducted between April and May 2013 employing Health Systems Availability Mapping System (HeRAMS). Data from 1581 primary care facilities were analysed, focusing on SRH services. Descriptive analyses and multivariable logistic regression models were used to examine the availability of SRH services by different levels of conflict and displacement.

**Findings:**

Of 1581 facilities, 1551 had data available to identify the details of service provision. The majority of the facilities were part of the public sector (79.1 %), identified as basic community primary care facilities (71.9 %). Overall 15.7 % of the facilities were in the zones under occupation, 40.3 % in the areas with high concentration of displaced population and 44 % in areas with low concentration of displaced populations. Between zones of low concentration of displaced populations and under occupation the likelihood of service availability varied between OR: 2.9 (95 % CI 2.0–4.4) for basic emergency obstetric care and OR: 41.7 (95 % CI 20.4–85.3) for family planning. All of the services within the three domains of SRH were more likely to be available in the low and high concentration displaced population areas compared to the facilities in the under occupation zones, after adjusting for other facility-related variables.

**Conclusion:**

Areas with high concentration of displaced population had less service availability, and areas formerly under occupation had the least service availability. This suggests that those living in conflict areas, and many of those who are internally displaced, have poor access to essential SRH interventions. The systematic measurement of the availability of health services, including SRH, is feasible and can contribute to recovery planning in post-conflict and humanitarian settings.

## Introduction

Although progress has been made in the past several decades in the recognition of the effect of conflict on the health of populations, much work remains to be done [11]. Sexual and reproductive health (SRH) in particular has been recognized as a key health issue for populations affected by conflict, yet it has often been under-prioritized [1]. Populations living in conflict-affected settings continue to need services for safe maternity care, family planning, newborn care, and other SRH needs, however adequate services are often not available in low-income settings even before a conflict. Afterwards, what little infrastructure existed to attend to the SRH needs of the population is often destroyed [6].

Little is known specifically about the effects of conflict and displacement on provision of SRH services. According to World Health Organization (WHO), key SRH interventions include: family planning, safe abortion care to the full extent of the law and post-abortion care, pregnancy care, childbirth care including emergency obstetric care, postnatal care (mother and newborn), prevention and management of sexually transmitted infections and HIV, including mother-to-child transmission of HIV and syphilis, prevention and management of gender-based violence [16]. Although some studies have shown increased vulnerability to sexually transmitted infections, including HIV, increased levels of gender-based violence, and worse birth outcomes in conflict-affected settings, the extent to which these findings from different countries experiencing different causes of conflict and displacement are generalizable is unclear [6]. A large, multi-country assessment found that although refugees living in camp settings had better access to reproductive health care than internally displaced or non-displaced populations, many still lacked access to life-saving emergency obstetric care or to a full spectrum of family planning options [14]. A more recent assessment of family planning service availability in the Democratic Republic of the Congo, Sudan and Northern Uganda similarly found that fewer than one-third of facilities which were expected to provide broad spectrum family planning services had the requisite supplies, staff, or equipment to meet the needs of potential clients, and in some regions no facilities were properly equipped [7].

**Fig. 1:**
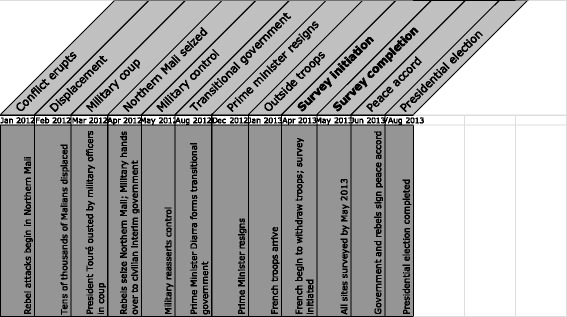
Timeline of events in Mali (January 2012–August 2013)

Mali is among the world's poorest countries [13], with one of the world's highest maternal mortality ratios at approximately 540 per 100,000 live births [22]. Against a backdrop of an ongoing crisis of food insecurity in the Sahel region, armed conflict broke out in Northern Mali in January 2012, leading to a divided country and internal and external displacement of its residents. Figure 1 describes the timeline of events. As of December 31, 2013, an estimated 422,900 Malians were displaced, the majority of whom (over 250,000) were displaced internally; and over three million Malians were facing moderate or severe food insecurity [15]. The conflict and ongoing insecurity led to physical destruction of health facilities, a lack of qualified health workers, and medical supply chain problems, particularly in the Northern conflict-affected areas [20].

A nationwide rapid assessment employing Health Systems Availability Mapping System (HeRAMS), further described below, was conducted by the World Health Organization (WHO) in partnership with the Ministry of Health (MoH) in post-conflict Mali in 2013. In this analysis, we aim to understand the association between levels of conflict and displacement and the availability of SRH services within the health sector in post-conflict Mali.

## Methods

HeRAMS survey was developed in response to the “lack of data for decision-making” during conflicts and humanitarian emergencies and mainly aims to systematically assess and monitor health services availability using a standardized tool within limited time, resources and accessibility. It has been used in a number of humanitarian settings, such as Haiti and Darfur [23]. The HeRAMS survey in Mali included all primary, secondary and tertiary health facilities registered under the MoH in the country except health structures such as pharmacies, labs and imaging facilities. It was conducted between April and May 2013 in Mali. This cross-sectional survey included questions on general information about the facility characteristics, accessibility, activity volume and questions related to the availability of specific health services under different domains such as child health, nutrition, communicable diseases and maternal and newborn health. More information on the HeRAMS, including the tool, approach and technical user guide [23] and the executive summary of the Mali HeRAMS survey are available online [17].

Briefly, the survey was initiated and adapted in consultation with the MoH Mali, the World Health Organization (WHO) country office and WHO’s Department of Emergency Risk Management and Humanitarian Response (ERM) and Global Health Cluster (GHC), and asked questions about the integrity of the facility and the health services that were being provided. The questionnaire was piloted in several health facilities in the Bamako region and adapted based on feedback. The survey was then sent to all primary, secondary and tertiary health facilities throughout the country, filled out by the social and health personnel at each facility Surveys were returned to the district offices after completion. In case of difficulty of access, the surveys were completed by telephone. Data entry was done by the WHO country office in Bamako, Mali. The modified HeRAMS tool and the protocol for Mali are available upon request.

Our analysis is limited to primary level facilities and focuses on services related to SRH. The following facility types were included: basic community primary care level (CSCOM), private primary level, public primary level reference (CSREF) facilities and others such as individual health cabinets, clinics and polyclinics. The SRH services were grouped under three categories: maternal and neonatal services, including family planning and comprehensive abortion care; services provided to the victims of sexual violence; and treatment for sexually transmitted infections and HIV/AIDS. Table 1 describes specific services and their operational definitions (where available), as used in HeRAMS Mali survey. We excluded secondary and tertiary level facilities in this analysis due to small number (*n* = 15) and the different mandate and range of services provided.

**Table 1:** The specific health services under maternal, sexual and reproductive health domains as defined in HeRAMS Mali, 2013

Maternal & Newborn Health
Family planning
Antenatal care: problems (assess pregnancy, birth and emergency plan, respond to problems (observed and/or reported), advise/counsel on nutrition & breastfeeding, self-care and family planning, preventive treatment(s) as appropriate.
Skilled care during childbirth: for clean and safe normal delivery
Essential newborn care: basic newborn resuscitation + warmth (recommended method: Kangaroo mother care) + eye prophylaxis + clean cord care + early and exclusive breastfeeding
Basic EmOC: parenteral antibiotics + oxytocic/anticonvulsant drugs + manual removal of placenta + removal of retained products with manual vacuum aspiration (MVA) + assisted vaginal delivery, 24 h 7 days a week
Comprehensive EmOC: Basic EmOC + ability to perform C-sections and blood transfusions
Postpartum care: examination of mother and newborn (up to 6 weeks), respond to observed signs, support breastfeeding, promote family planning
Comprehensive abortion care: safe induced abortion for all legal indications, uterine evacuation using MVA or medical methods, antibiotic prophylaxis, treatment of abortion complications, counselling for abortion and post-abortion contraception
Sexual Violence
Clinical management of rape survivors (including psychological support)
Emergency contraception
Post-exposure prophylaxis for STI & HIV infections
STI & HIV/AIDS
Syndromic management of STIs
Standard precautions: disposable needles & syringes, safety sharp disposal containers, personal protective equipment, sterilizer
Availability of free condoms
Prophylaxis and treatment of opportunistic infections (HIV/AIDS)
HIV counseling and testing
Prevention of maternal-to-child transmission of HIV (PMTCT)
Anti-retroviral treatment for HIV (ARV)

Ethical review was not required for this survey as data collection did not include direct interaction with human subjects.

### Variables

**Fig. 2:**
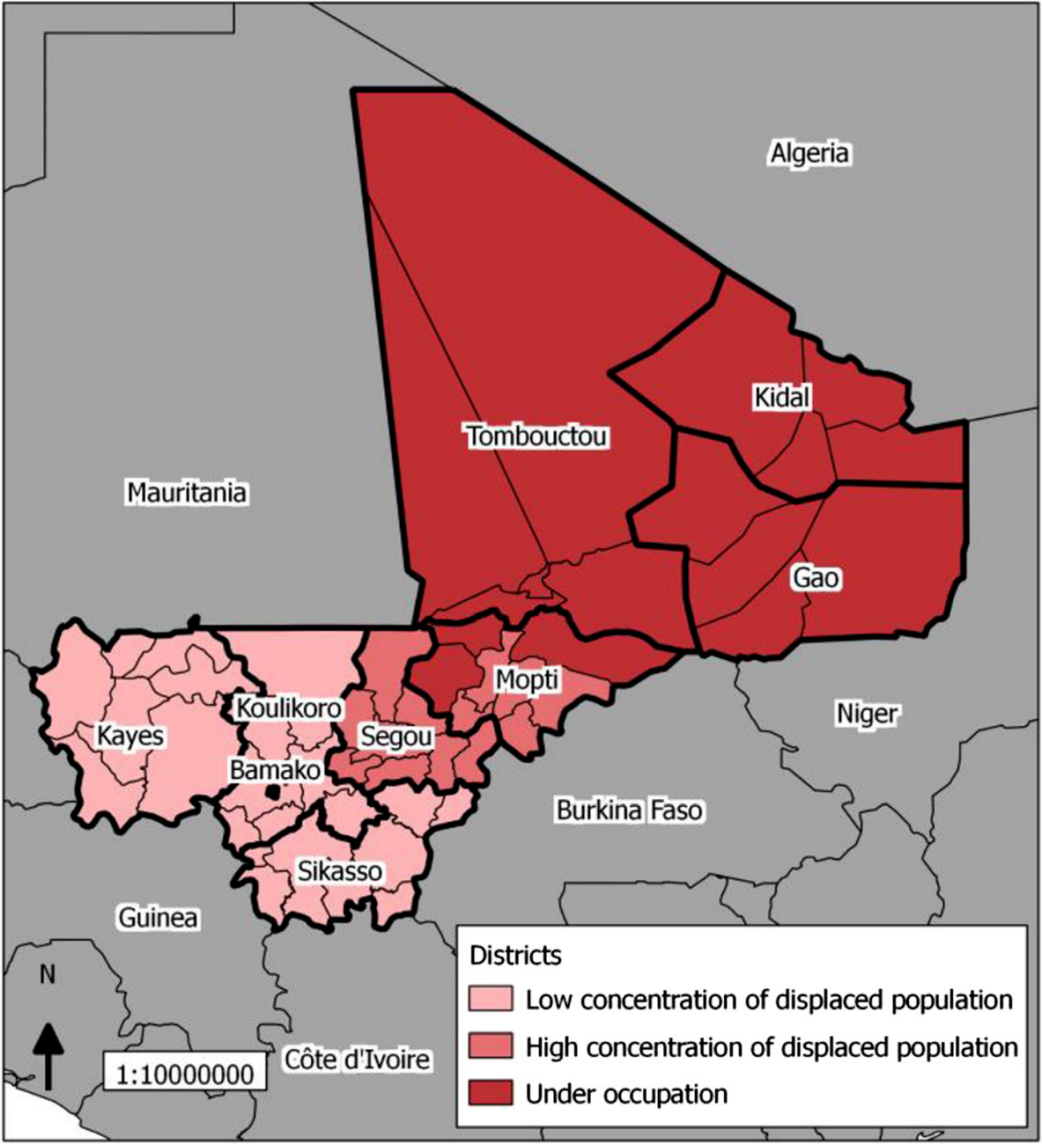
Distribution of districts by levels of conflict, February 2013, reproduced based on [4, 18]

The variables used in this analysis were created using the quantitative responses to the survey questions. According to the MoH Mali, each region was assigned a level of conflict and displacement and designated as either under occupation, or having a high concentration of displaced population, or having a low concentration of displaced population, based on its status between January 2012 and January 2013 [17, 18] (Fig. 2). As described in the protocol, the towns occupied by the armed groups were identified as under occupation and levels of displacement were determined by the government based on the number of internally displaced persons (IDP) and IDP camps [4, 18].

The main outcome variable was the availability of services, defined in two stages. First, we relied on self-report by the facilities to determine whether the service fell within the facility's mandate. Among those facilities that reported a mandate to provide the service, services were coded as “available” if the response was "perfectly satisfactory" or "satisfactory". Primary level facilities (of which there are several types in Mali) were coded according to the categories used by the MoH. Each facility was assessed and categorized using two separate questions from the survey on functionality (non-functioning/functioning) and physical status of the facility (non-intact/intact) as reported by the facility personnel who completed the surveys. If a facility was both functional and intact, it was coded as functional/intact and if a facility was either non-functioning and/or non-intact, it was coded as non-functional and/or non-intact. Other variables used were sector of the facility as registered under the government (private or public) and external support to the facility by non-governmental organizations, United Nations organizations or others defined as “partial or complete support” or “no external support”. Water availability was assessed as "Good access" if the principal water source was reported as “tap water”, “borehole” or “protected well;” otherwise, it was coded as “No access”.

### Statistical analysis

Using descriptive analyses and multivariable logistic regression models, we examined the availability of reproductive health services by different levels of conflict and displacement (under occupation, high concentration of displaced population and low concentration of displaced population). In our models, under occupation has been used as the reference group.

Facilities were asked whether they had the mandate to provide each specific service for all of the domains. Based on the descriptive analyses, facilities reporting a specific service within their mandate were included in the logistic regression analyses. Additionally, services that more than 50 % of facilities reported as “not planned to be provided” were excluded from further analyses.

In the multivariable logistic regression analyses, the models were adjusted for variables that were identified *a priori* which could influence the availability of services: the sector (public or private), external support to the facility, physical status evaluation and access to water. Crude odds ratios and adjusted odds ratios (AOR) were reported. Models were checked for fitness and collinearity. All analyses were conducted using Stata SE Version 12, StataCorp LP, Texas, USA.

## Results

Data from 1581 primary care facilities were analysed based on characteristics of these facilities (Table 2). The majority of the facilities were part of the public sector (79.1 %), identified as basic community primary care facilities (CSCOM) (71.9 %), functional and intact (72.2 %) and with access to water (76.7 %). It should be noted that 45.8 % of the facilities received partial support, whereas only 117 (7.5 %) received total support from partners. The majority of this support came from non-governmental organizations (NGOs). More than half of the facilities were located in very poor areas, as defined by the Government of Mali. Free care for displaced persons was reported to be available only in 163 facilities (10.3 %). Overall 15.7 % of the facilities were in the zones under occupation, 40.3 % in the areas with high concentration of displaced population and 44 % in areas with low concentration of displaced populations.

**Table 2:** Characteristics of the primary care facilities in Mali (*N* = 1,581)

	Low concentration of displaced population	High concentration of displaced population	Under occupation	Total
	*N* = 706	*N* = 627	*N* = 248	*N* = 1581
	n (%)	n (%)	n (%)	n (%)
Facility type				
Basic primary level – community (CSCOM)	593 (84.0)	342 (54.5)	202 (81.4)	1137 (71.9)
Primary level - private	42 (6.0)	146 (23.3)	3 (1.2)	191 (12.1)
Primary level reference – public (CSREF)	25 (3.5)	19 (3.1)	16 (6.5)	60 (3.8)
Other^a^	46 (6.5)	120 (19.1)	27 (10.9)	193 (12.2)
Sector				
Public	624 (88.4)	380 (60.6)	246 (99.2)	1250 (79.1)
Private	82 (11.6)	247 (39.4)	2 (0.8)	331 (20.9)
External support to the facility				
No external support	269 (38.1)	343 (54.7)	112 (45.2)	724 (45.8)
Partial or complete external support	436 (61.8)	284 (45.3)	108 (43.6)	828 (52.4)
Missing	1 (0.1)	0 (0.0)	28 (11.3)	29 (1.8)
Evaluation of the facility				
Non-functioning and/or non-intact	160 (22.7)	100 (16.0)	175 (70.6)	435 (27.5)
Functional and intact	543 (76.9)	527 (84.0)	72 (29.0)	1142 (72.2)
Missing	3 (0.4)	0 (0.0)	1 (0.4)	4 (0.3)
Access to water				
No access	179 (23.4)	78 (12.4)	82 (33.1)	339 (21.5)
Access	526 (74.5)	549 (87.6)	138 (55.6)	1213 (76.7)
Missing	1 (0.1)	0 (0.0)	28 (11.3)	29 (1.8)
Free health care for displaced population				
No	648 (91.8)	522 (83.2)	248 (100.0)	1418 (89.7)
Yes	58 (8.2)	105 (16.8)	0 (0.0)	163 (10.3)

^a^Other refers to individual health cabinets, clinics and polyclinics

Of 1581 facilities, 1551 had data available to identify the details of service provision, as shown in Table 3. Thirty facilities with missing information on service availability were excluded as follows: Kidal (15), Gao (7), Tombouctou (4), Mopti (2), Bamako (1), Koulikoro (1). The percentage of facilities reporting on each service as “not planned to be provided” at the individual facility ranged from 7.2 % for family planning to 33 % for clinical management of rape survivors and 39 % for prophylaxis and treatment of opportunistic infections. As described in our methodology, the services where more than 50 % of the primary level facilities reported as outside of their mandate have been excluded from further analyses. These include comprehensive emergency obstetric care (EmOC) (85.2 %), availability of free condoms (56.4 %) and antiretroviral treatment for HIV (63.8 %). It should be noted that we further explored the comprehensive EmOC availability by facility type and discovered that 91 % of both the private and community primary care facilities (CSCOM) reported this service out of their mandate, whereas only 9 % of the sixty public primary level reference facilities (CSREF) reported not being mandated to provide comprehensive EmOC. This distribution was not influenced by the conflict and occupation levels. Overall, the percentage of facilities sufficiently providing services as planned varied by service. For example, for maternal and newborn health services, it ranged between 66 % for basic EmOC and 93 % for antenatal care [9].

**Table 3:** Availability of sexual and reproductive health services in the primary care facilities (*N* = 1,551)

	Not available at the time of the survey	Available at the time of the survey	Not planned to be provided at the facility	% of facilities sufficiently providing services as planned
	n (%)	n (%)	n (%)	% (n/N)
Maternal & Newborn Health				
Family planning	111 (7.2)	1329 (85.7)	111 (7.2)	92 (1329/1440)
Antenatal care	97 (6.2)	1330 (85.8)	124 (8.0)	93 (1330/1427)
Skilled care during childbirth	148 (9.5)	1273 (82.1)	130 (8.4)	89 (1273/1421)
Essential newborn care	141 (9.1)	1236 (79.7)	174 (11.2)	90 (1236/1377)
Basic EmOC	341 (22.0)	660 (42.6)	550 (35.5)	66 (660/1001)
Comprehensive EmOC	103 (6.6)	126 (8.1)	1322 (85.2)^a^	55 (126/229)
Postpartum care	102 (6.6)	1277 (82.3)	172 (11.1)	93 (1277/1379)
Comprehensive abortion care	301 (19.4)	676 (43.6)	574 (37.0)	69 (676/977)
Sexual Violence				
Clinical management of rape survivors	325 (20.9)	715 (46.1)	511 (33.0)	69 (715/1040)
Emergency contraception	373 (24.1)	682 (43.9)	511 (33.0)	65 (682/1055)
Post-exposure prophylaxis for STI & HIV infections	314 (20.2)	814 (52.5)	423 (27.3)	72 (814/1128)
STI & HIV/AIDS				
Syndromic management of STIs	126 (8.1)	1290 (83.2)	135 (8.7)	91 (1290/1416)
Standard precautions	94 (6.1)	1354 (87.3)	103 (6.6)	94 (1354/1448)
Availability of free condoms	356 (22.9)	321 (20.7)	874 (56.4)^a^	47 (321/677)
Prophylaxis and treatment of opportunistic infections (HIV/AIDS)	289 (18.6)	651 (42.0)	612 (39.4)	69 (651/940)
HIV counseling and testing	386 (24.9)	708 (45.6)	457 (29.5)	65 (708/1094)
Prevention of maternal-to-child transmission of HIV (PMTCT)	501 (32.3)	455 (29.3)	595 (38.4)	48 (455/956)
Anti-retroviral treatment for HIV (ARV)	363 (23.4)	199 (12.8)	989 (63.8)^a^	35 (199/562)

^a^The services where more than 50 % of the primary level facilities reported as outside of their mandate have been excluded from further analyses

Table 4 shows the results of both the unadjusted and adjusted models assessing the availability of services according to the level of conflict and displacement, where facilities located in regions with either a low or a high concentration of displaced persons are compared with facilities in areas under occupation. The results show that all of the services are more likely to be available in those areas with displaced populations compared to those in the zones under occupation. Compared to zones under occupation the likelihood of service availability was significantly higher in the zones of low concentration of displaced populations and varied between OR: 2.9 (95 % CI 2.0–4.4) for basic emergency obstetric care and OR: 41.7 (95 % CI 20.4–85.3) for family planning.

**Table 4:** Unadjusted and adjusted^a^ multivariable logistic regression analyses assessing the association between availability of reproductive health services and level of conflict and displacement (Reference group: Under occupation)

		High concentration of displaced population		Low concentration of displaced population	
	N	Crude OR (95 % CI)	Adjusted OR (95 % CI)	Crude OR (95 % CI)	Adjusted OR (95 % CI)
Maternal & Newborn Health					
Family Planning	1440	11.6 (7.2–18.9)^***^	12.9 (5.8–28.9)^***^	41.7 (20.4–85.3)^***^	30.7 (13.5–69.6)^***^
Antenatal care	1424	14.5 (8.5–24.9)^***^	22.7 (8.2–62.8)^***^	56.9 (24.2–133.5)^***^	38.6 (14.9–99.9)^***^
Skilled care during childbirth	1418	5.1 (3.4–7.7)^***^	1.8 (1.1–3.0)^*^	10.9 (6.8–17.5)^***^	4.9 (3.0–8.2)^***^
Essential newborn care	1374	8.7 (5.7–13.3)^***^	10.3 (5.6–18.9)^***^	32.4 (18.1–58.2)^***^	24.7 (13.2–46.4)^***^
Basic EmOC	999	5.6 (3.7–8.4)^***^	2.9 (1.8–4.6)^***^	2.9 (2.0–4.4)^***^	2.1 (1.4–3.2)^**^
Postpartum care	1376	15.3 (8.7–26.9)^***^	15.8 (6.8–36.3)^***^	16.7 (9.7–28.8)^***^	10.1 (5.5–18.6)^***^
Comprehensive abortion care	975	12.7 (8.0–20.2)^***^	6.3 (3.8–10.4)^***^	5.1 (3.3–7.9)^***^	3.6 (2.2–5.7)^***^
Sexual Violence					
Clinical management of rape survivors	1039	6.0 (3.8–9.6)^***^	4.6 (2.7–7.7)^***^	4.8 (3.0–7.6)^***^	4.4 (2.7–7.3)^***^
Emergency contraception	1053	7.0 (4.5–11.1)^***^	2.9 (1.8–4.9)^***^	4.3 (2.7–6.8)^***^	2.6 (1.6–4.3)^***^
Post-exposure prophylaxis for STI & HIV infections	1125	6.5 (4.3–9.6)^***^	3.7 (2.3-5.9)^***^	4.0 (2.8–5.8)^***^	2.4 (1.6–3.7)^***^
STI & HIV/AIDS					
Syndromic Management of STIs	1413	10.0 (6.3–15.8)^***^	11.3 (5.6–22.5)^***^	16.6 (9.9–27.8)^***^	10.7 (5.9–19.1)^***^
Standard precautions	1445	8.3 (4.9–14.1)^***^	5.1 (2.5–10.5)^***^	12.8 (7.3–22.5)^***^	7.1 (3.8–13.0)^***^
Prophylaxis and treatment of opportunistic infections (HIV/AIDS)	937	4.3 (2.8–6.5)^***^	3.3 (2.0–5.3)^***^	5.3 (3.5–7.9)^***^	3.3 (2.0–5.3)^***^
HIV counseling and testing	1092	7.7 (5.1–11.6)^***^	3.1 (1.9–5.0)^***^	7.5 (5.0–11.4)^***^	4.3 (2.7–6.7)^***^
PMTCT	954	7.0 (4.5–11.1)^***^	3.7 (2.2–6.1)^***^	4.0 (2.6–6.3)^***^	2.5 (1.5–4.1)^***^

^*^ *p* < 0.05; ^**^ *p* < 0.01;^***^ *p* < 0.001

^a^ Models were adjusted for sector (public/private), external support to the facility, evaluation of the facility (function/intact) and access to water

In the adjusted model, almost all of the associations between levels of conflict and displacement and availability of the services attenuated. However all of the services within the three domains of SRH were still more likely to be available in the low and high concentration displaced population areas compared to the facilities in the under occupation zones, after adjusting for other facility related variables. Compared to facilities located in regions under occupation, skilled care during childbirth was significantly more likely to be available in the facilities of regions with high and low concentration of displaced populations, AOR: 1.8 (95 % CI: [1.1–3.0]) and AOR: 4.9 (95 % CI: [3.0–8.2]) respectively. Rape survivors were more likely to find available services outside of the occupation regions, AOR: 4.6 (95 % CI: [2.7–7.7]) in regions with high concentration of displaced populations and AOR: 4.4 (95 % CI: [2.7–7.3]) in regions with low concentration of displaced population, compared to the areas under occupation. Again, the likelihood of finding a facility with HIV counselling and testing was significantly higher in the rest of the country outside of the occupation zones, AOR: 3.1 (95 % CI: [1.9–5.0]) in regions of high concentration of displaced population and AOR: 4.3 (95 % CI: [2.7–6.7]) in regions with a low level of displaced population.

The associations with other variables regarding the functionality, sector and support to the facility differed for services (results not shown). Of note, a number of services were more likely to be available in the private facilities: Basic EmOC (AOR: 2.2, 95 % CI [1.4–3.5]), comprehensive abortion services (AOR: 2.5, 95 % CI [1.5–4.3]), clinical management of rape survivors (AOR: 3.0, 95 % CI [1.9–4.9]), emergency contraception (AOR: 3.8, 95 % CI [2.3-6.1]), HIV counselling and testing services (AOR: 3.5 (95 % CI [2.2–5.6]) and PMTCT (AOR: 1.6 (95 % CI [1.1–2.4]). Physical and functioning status of the facility was consistently positively associated with the availability of the services across all the domains. Partial or complete external support to the facility was in most cases associated with a greater likelihood of providing services, with the exception of BEmOC and comprehensive abortion care as well as prophylaxis and treatment of opportunistic infections - where the associations were non-significant.

## Discussion

To the best of our knowledge, this is the first national level analysis that has assessed SRH services in all primary health facilities in a post-conflict setting and analysed service availability based on levels of conflict and displacement. Our analysis suggests that levels of conflict and displacement are associated with availability of critical SRH services. Although almost all these associations attenuated in the multivariable logistic models, they still remained significant. Multivariable analyses showed that, in general, services were most likely to be available in areas with low concentration of displaced population. As can be predicted, areas with high concentration of displaced population had less service availability, and areas under occupation had the least service availability. This suggests that those living in conflict areas, and many of those who are internally displaced, have poor access to essential SRH interventions. Epidemiology-based activities, such as HeRAMS, conducted during and after an emergency event contribute to better understanding of the situation, needs and provide actionable information [5]. Therefore, it will be essential to keep improving the HeRAMS survey by examining and aligning the questions, indicators and their definitions, where possible, with other relevant and/or applicable tools such as Service availability and Readiness Assessment (SARA) and Balance Scorecard to facilitate the continuity and comparability of monitoring outside of emergencies [8, 10, 24].

There are a number of limitations which need to be highlighted. This was a cross-sectional study based on self-report, in the context of ongoing population movement and evolving post-conflict situation and only represents one point in time, therefore our results can only signify an association rather than causality. Furthermore, we do not have data on availability of SRH services before the conflict and the regions under occupation and/or with high concentration of displaced population might have less availability of services to begin with. In our models, residual confounding is a likely problem, as we only included the variables which were readily available in the survey, and there might be other variables influencing the relationship between level of conflict and availability of services which we have not accounted for, such as availability of the commodities, health workforce. Moreover service availability is assessed by self-report, not by direct observation, which may have led to a degree of misclassification. It should be noted that due to the relatively smaller number of facilities in the reference group (facilities in the occupation zones) the confidence intervals are larger around the odds ratios.

SRH is a significant public health need in all communities, including those facing humanitarian emergencies [16]. Despite multiple international standards defining reproductive health care as a right and essential component of humanitarian response [3, 12], and WHO policies [16] - during conflict evidence-based interventions such as basic emergency obstetric care, essential newborn care, and clinical management of rape were not routinely available at the primary health care level in Mali. It was observed that partial or complete external support to the facility was in most cases associated with a greater likelihood of providing SRH services, with the notable exceptions of basic EmOC and comprehensive abortion care. Moreover private facilities were significantly less likely to offer some services such as antenatal care and syndromic management of STIs; this is likely because many private facilities are specialized centres, rather than more integrated primary care facilities. This underscores the importance of taking a proactive approach in strengthening SRH services in prevention of and preparation for humanitarian emergencies while supporting national and local authorities as well as communities. Moreover, even though we did not have a pre-conflict baseline assessment, our results underline the importance of the humanitarian response and support where we found that partial or complete external support to the facilities by NGOs, UN organizations and others was associated with a greater likelihood of providing SRH services. Though, stand-alone NGO operations providing services in the regions were not captured by this survey.

It should be noted that having the infrastructure is important yet does not equate to availability of services. HeRAMS Mali survey found that nearly one in five health facilities nationally was at least partially damaged. Significant regional disparities were noted, with over 40 % of facilities at least partially damaged in the Northern regions [17]. In terms of the main reasons for non-coverage of key services such as general clinical services, child health, communicable diseases and SRH, more so than infrastructure, lack of qualified medical staff and commodities including medical devices and medications were reported throughout the country, and was most problematic in the North [17].

In broader context of health systems, the conflict in Mali has impacted all WHO health system building blocks (service delivery, healthcare workforce, information, medical products/vaccines/technologies, financing and leadership and governance), and depending on the level of conflict and displacement, requiring a tailored approach for response [2, 19]. Based on the results of the 2013 HeRAMS, the following priorities relevant to SRH were identified for action: “Strengthening of the health system in the southern regions in order to cope with the influx of internally displaced persons, regular provision of medicines and other medical supplies, resumption of activities in destroyed, looted or vandalized health centres and hospitals which are still affected by staff shortages, the lack of qualified staff and the interruption of health programmes, restoration of referral mechanisms for medical, obstetric-surgical emergencies, especially where there is a lack of qualified staff, medicines and medical supplies, and where water pumps and electrical installations in health centres have been destroyed” [19]. In response, the Health Cluster -bringing together UN agencies, technical services and NGOs under the leadership of WHO and the National Health Directorate-, coordinated the health interventions and ensured that the humanitarian missions were organized to assure the continuity of health care provision in the districts with the most need and over 450 health workers were mobilized, a number of health facilities were re-opened and availability of services have improved [19]. In line with the Granada Consensus on Sexual and Reproductive Health in Protracted Crises and Recovery, which calls for mainstreaming SRH in health system revitalization and sustainable consolidation and expansion of SRH services based on local needs and context, our analysis further highlights the importance of attention given to the range of SRH services provided in the facilities in a post-conflict setting [21].

It is important to underline that demand for facility-based services may decrease in environments where there has been political instability, thus rebuilding trust in the health system may be as important as rebuilding facilities and improving which services are available [3]. It has been said that “measurement is visibility” and surveys such as HeRAMS Mali demonstrates systematic measurement of the availability of health services, including SRH, is feasible and can contribute to recovery planning in post-conflict and humanitarian settings.
